# The predictive value of D-dimer test for venous thromboembolism during puerperium in women age 35 or older: a prospective cohort study

**DOI:** 10.1186/s12959-020-00241-y

**Published:** 2020-10-16

**Authors:** Wen Hu, Dong Xu, Juan Li, Cheng Chen, Yuan Chen, Fangfang Xi, Feifei Zhou, Xiaohan Guo, Baihui Zhao, Qiong Luo

**Affiliations:** Department of Obstetrics, Women’s Hospital, Zhejiang University, School of Medicine, 1st Xueshi Road, Hangzhou, 310006 Zhejiang China

**Keywords:** D-dimer, Venous thromboembolism, Women age at 35 years or older, Puerperium

## Abstract

**Background:**

This study aimed to investigate the predictive value of the D-dimer level for venous thromboembolism (VTE) events during puerperium of women age at 35 years or older, as well as to identify other risk factors associated with the occurrence of VTE.

**Methods:**

It was a prospective observational cohort study, from January 2014 to December 2018, which involved 12,451 women age 35 or older who delivered at least 28 weeks of gestation at Women’s Hospital of Zhejiang University, School of Medicine. The maternal and fetal demographic characteristics, pregnancy complications, imaging finding and results of laboratory test within postpartum 24 h including D-dimer level, platelet counts and fibrinogen level were collected for analyses.

**Results:**

30(2.4‰) women were identified as VTE, including 1 pulmonary embolism event and 29 deep venous thrombosis events. The receiver operating characteristic (ROC) curve analysis suggested the best cutoff point for D-dimer level within postpartum 24 h of women age 35 or older was 5.545 mg/L, with a specificity of 70.0% and a sensitivity of 75.4%. Besides, there was no statistical correlation between platelet counts and VTE, as well as between fibrinogen level and VTE. On multivariate analysis, D-dimer≥5.50 mg/L (OR = 5.874, 95%CI: 2.678–12.886) and emergency cesarean section (OR = 11.965, 95%CI: 2.732–52.401) were independently associated with VTE in puerperium of women age 35 or older.

**Conclusions:**

We concluded that D-dimer≥5.50 mg/L was an independent predictor of VTE in puerperium with maternal age 35 or older and D-dimer testing was a necessary examination for perinatal women.

## Background

Venous thromboembolism (VTE) remains one of the leading causes of maternal mortality [1], taking the place of postpartum hemorrhage, which has been highly prevented and treated. The incidence is 4 to 5 times higher among pregnant and postpartum women than that of non-pregnant women [2]. Pregnancy is an acquired and independent risk factor for the development of VTE. Many other risk factors have been linked to VTE, such as advanced maternal age, thrombophilia, cesarean section, obesity, and a personal or family history of VTE [3, 4]. In recent years, maternal age at childbirth continues to increase worldwide, particularly in China, as a consequence of the changes in attitudes towards fertility and the adjustment of the birth policy. Increasing maternal age is associated with the increasing incidence of pregnancy complications, which together lead to the increasing incidence of VTE.

Among the screening tools for VTE, D-dimer testing has been proved its reliability in non-pregnant individuals by several studies, with high sensitivity and moderate specificity [5]. For pregnant women, D-dimer concentration increased progressively during the pregnancy and peaked at the first postpartum day [6]. Some studies proved the predictive value of D-dimer test for pregnant related VTE by raising the cutoff value or finding a higher D-dimer reference range [6–8]. D-dimer level also has been shown to increase by patient age [9]. However, there is a lack of research on the predictive value of D-dimer level for VTE in the women age 35 or older.

Therefore, we designed a prospective observational study to identify the incidence and risk factors of VTE during the postpartum period in women age 35 or older. Furthermore, we investigated the predictive value of coagulation markers including D-dimer level, platelet counts and fibrinogen level, and attempted to determine a suitable threshold for the assessment in postpartum period of older mothers.

## Methods

### Patients

Women’s Hospital, School of Medicine, Zhejiang University (WHZJU) has 460 maternity beds and serves many provinces of East China region. Approximately 20,000 births occur annually. As a first-class specialized hospital of obstetrics and gynecology in China and a nationally-known referral center, many of the pregnant women are complicated and high-risk.

**Fig. 1:**
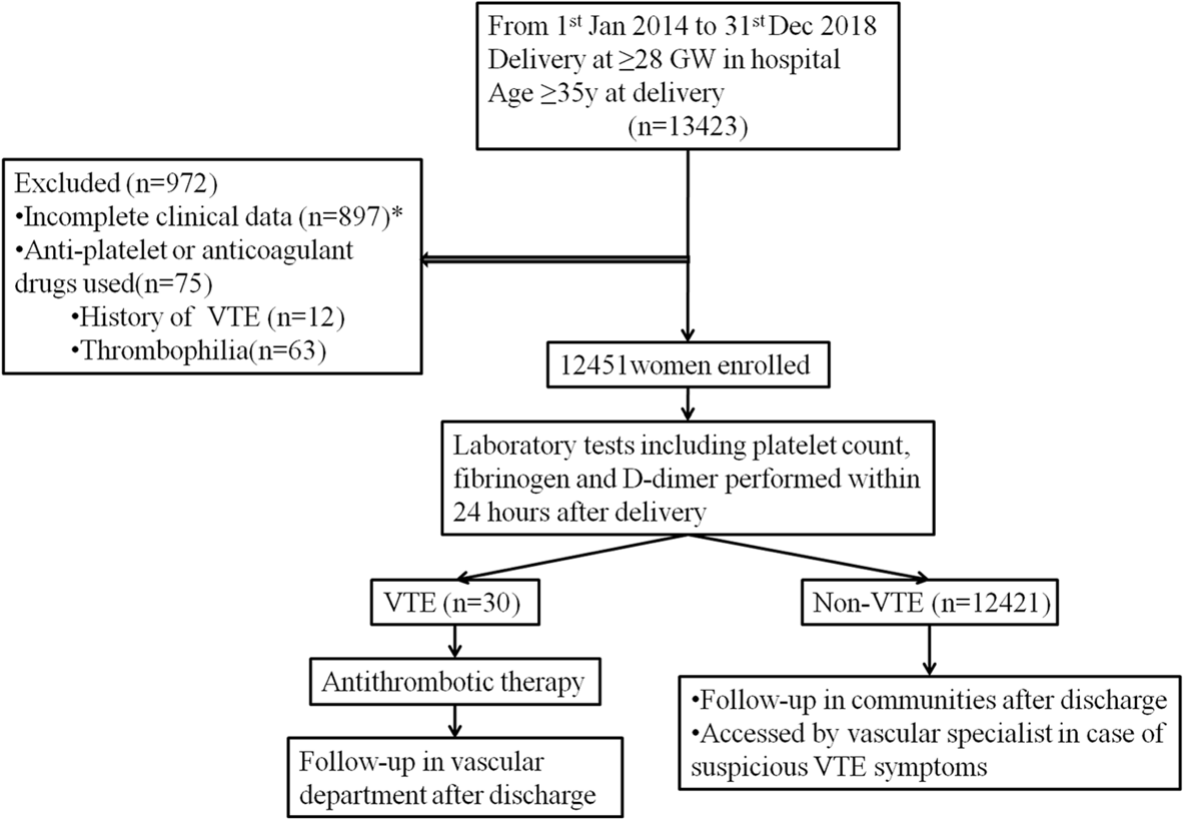
Flow chart of indicating the patients included in and excluded from the study. * Lack of blood test data within 24 h after delivery because of delayed detection or patients’ unwillingness to participate

We initiated the study in January 1st 2014 and continued until December 31st 2018, and we prospectively collected the data of women age 35 or older who gave birth at least 28 weeks of gestation at WHZJU. Women used anticoagulant or anti-platelet drugs before delivery or with incomplete clinical data were excluded from this study (Fig. 1). The sample size required for the study was calculated according to the following formula:

$$ \mathrm{n}=\frac{{\left({\mathrm{Z}}_{\upalpha}\sqrt{2\mathrm{pq}}+{\mathrm{Z}}_{\upbeta}\sqrt{{\mathrm{p}}_0{\mathrm{q}}_0+{\mathrm{p}}_1{\mathrm{q}}_1}\right)}^2}{{\left({\mathrm{p}}_1-{\mathrm{p}}_0\right)}^2}. $$

In this formula, p_0_ indicated the incidence of VTE in D-dimer< 5.545 mg /L group (0.96‰); p_1_ indicated the incidence of VTE in D-dimer≥5.545 mg /L group (6.8‰); p stood for the average value of p_0_ and p_1_; q = 1-p. We set α = 0.05 and β = 0.10 (power = 0.90); z_α_ = 1.96 and z_β_ = 1.282, which represented for the boundary value of normal distribution. The estimated sample size was 2380. The sample size of this study was larger than the estimated sample size. All clinical variables were recorded, including age, body mass index (BMI), pregnancy times, parity, gestational weeks of delivery, fetal position, mode of delivery, fetal birth weight, pregnancy complications, postpartum hemorrhage and predictive biomarkers within postpartum 24 h including D-dimer level, platelet counts and fibrinogen level. All biomarker values were obtained from the same laboratory affiliated to the hospital.

### Clinical diagnosis of VTE

Imaging evidence was confirmed as the diagnostic criteria for VTE. Deep venous thrombosis (DVT) was diagnosed by upper and lower extremity venous color Doppler ultrasound and/or computed tomographic (CT) venography, and pulmonary embolism (PE) was diagnosed by CT pulmonary angiography.

Imaging examinations were required if the following conditions were present: (1) with suspicious symptoms of VTE, including pain or tenderness when move limbs, swelling of the limbs, measurement of inconsistencies in the circumference of the bilateral limbs, or unexplained dyspnea, chest pain or cough; or (2) with multiple high risk factors, and the clinician considered that the probability for VTE was great. Anticoagulation and antithrombotic therapy would be applied immediately when imaging examination indicated the diagnosis of VTE. All the VTE patients were told to follow up in the vascular department after discharge. Other women were followed up in the communities and would be accessed by vascular specialist in case of suspicious VTE symptoms (Fig. 1).

### Laboratory assays

We performed laboratory tests including platelet counts, fibrinogen level and D-dimer level. The detection of platelet counts was measured by impedance (XN9000; Sysmex, Kobe, Japan). The detection of fibrinogen level was measured by the solidification (Stago-R, Paris, France). The detection of D-dimer level was measured by the latex-enhanced immunoturbidimetry (Stago-R, Paris, France) (normal reference range for non-pregnant adults is less than 0.5 mg/L).

### Statistical analyses

Data inputting and statistical analysis were performed in SPSS 22.0 (IBM Corporation, New York, USA). Continuous variables were described as means ± standard deviation. The continuous variables were compared by Student’s T test. The difference in the categorical variables was compared through Chi square test, Yate’s correction of continuity or Fisher’s exact test. Furthermore, to estimate the risk factors of VTE, the forward stepwise multiple logistic regression was performed. The associations between biomarkers and VTE were expressed as ROC curve analysis. Statistical significance was set at *p* < 0.05.

## Results

Twelve thousand four hundred fifty-one women were enrolled in this study after screening (Fig. 1). In our cohort, 30 (2.4‰) women were identified as VTE, including 1 PE event and 29 DVT events. The DVT events included: 5 women with bilateral DVT of lower extremity, 11 women with DVT of right lower extremity and 13 with DVT of left lower extremity. VTE occurred at median of 3.5 days postpartum (range: 2–15 days).

**Fig. 2:**
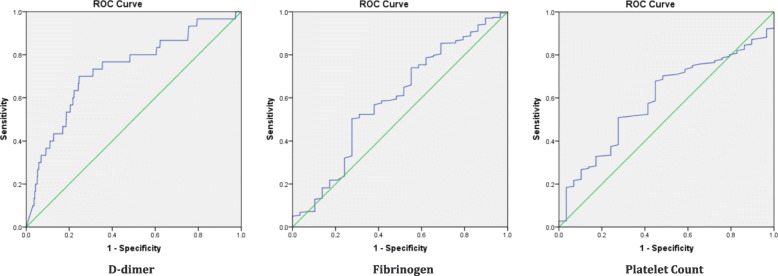
Receiver operating characteristic (ROC) curve for D-dimer, fibrinogen, and platelet count within postpartum 24 h of women age ≥ 35. AUC (ROC of D-dimer): 0.732 (*P* < .001); AUC (ROC of fibrinogen): 0.592 (*P* = .086); AUC (ROC of platelet count): 0.594 (*P* = .081)

All the D-dimer test results of VTE patients in this study exceeded the upper limit of the reference value (0.5 mg/L). The ROC curve analysis showed the best cutoff point for D-dimer level within postpartum 24 h of women age 35 or older was 5.545 mg/L, with a specificity of 70.0% and a sensitivity of 75.4%(Fig. 2). For convenience in clinical practice, the predefined cutoff value for dichotomized variables of D-dimer level was set at 5.50 mg/L. When the cutoff value was set at 6.475 mg/L, the specificity could increase to 80.0%, but the sensitivity would decrease to 53.3%; when the cutoff value was set at 9.875 mg/L, the specificity could increase to 90.0%, but the sensitivity would decrease to 36.7%.The AUCs of fibrinogen level and platelet counts were close to 0.5, indicating that there was no statistical correlation between them and VTE (Fig. 2).

Table 1 shows a comparison of maternal and fetal characteristics between VTE and non-VTE groups. Age, heights, gestational weeks of delivery, neonatal weight, fibrinogen level and platelet counts were not significantly associated with VTE. The average D-dimer level within postpartum 24 h with maternal age ≥ 35 in VTE group was significantly higher than that of non-VTE group (8.91 vs. 4.55 mg/L, *P* < 0.001).

**Table 1:** Comparison of general characteristics between VTE and non-VTE delivery women age ≥ 35(x ± SD)

	VTE (*n* = 30)	Non-VTE (*n* = 12,421)	*t* value	*P* value
Age (years)	38.10 ± 2.23	37.31 ± 2.29	1.893	.058
Height (cm)	161.13 ± 5.76	160.57 ± 4.71	0.650	.516
BMI before pregnancy	21.65 ± 2.96	21.05 ± 2.91	1.139	.255
BMI before delivery	26.70 ± 2.80	26.50 ± 2.99	0.363	.717
Gain weight during pregnancy (kg)	13.07 ± 3.19	14.04 ± 4.94	−1.067	.286
Gestational age at delivery (weeks)	37.83 ± 2.26	37.99 ± 1.95	−0.447	.655
Neonatal birth weight(g)	3114.67 ± 558.19	3203.16 ± 604.89	−0.800	.423
Laboratory test results within postpartum 24 h				
D-dimer (mg/L)	8.91 ± 6.16	4.55 ± 4.31	3.870	.001
Fibrinogen (g/L)	4.37 ± 0.85	4.63 ± 0.81	−1.706	.088
Platelet count (10^9/L)	172.00 ± 40.50	187.82 ± 53.53	−1.590	.112

The risk factors predisposing to VTE in puerperium of women age ≥ 35 were analyzed in Table 2. Mode of delivery, scared uterus, and D-dimer≥5.50 mg/L were significantly associated with VTE in puerperium of older mothers (Table 2).

**Table 2:** Risk factors predisposing to VTE in puerperium of women age ≥ 35

Risk factors	VTE(%) (*n* = 30)	Non-VTE(%) (*n* = 12,421)	*χ*^*2*^ value	*P* value
Previous obstetric history				
0	11 (36.67)	2938 (23.65)	2.804	.094
≥ 1	19 (63.33)	9483 (76.35)		
Parity				
1	30 (100.00)	12,048 (97.00)		1.000^a^
≥ 2	0 (0.00)	373 (3.00)		
Fetal position of singleton cases				
Head	28 (93.33)	11,406 (94.67)		.385^a^
Breech	1 (3.33)	506 (4.20)		
Transverse	1 (3.33)	136 (1.13)		
Mode of delivery				
Vaginal delivery	2 (6.67)	4786 (38.53)	25.792	<.001
Emergency cesarean section	16 (53.33)	2399 (19.31)		
Elective caesarean section	12 (40.00)	5236 (42.15)		
In vitro fertilization (IVF)	0 (0.00)	678 (5.46)	0.834	.361
Scared uterus	20 (66.67)	5612 (45.18)	5.58	.018
Relative cephalopelvic disproportion	0 (0.00)	164 (1.32)		1.000^a^
Placenta previa	3 (10.00)	612 (4.93)	0.738	.390
Adherent placenta	4 (13.33)	610 (4.91)	2.910	.088
Fetal growth restriction	0 (0.00)	182 (1.47)		1.000^a^
Premature birth	5 (16.67)	1557 (12.54)	0.165	.684
Macrosomia (birthweight≥4000 g)	1 (3.33)	789 (6.35)	0.092	.762
Premature rupture of membranes	7 (23.33)	2184 (17.58)	0.682	.409
Fetal distress	5 (16.67)	1566 (12.61)	0.155	.694
Intrauterine infection	1 (3.33)	139 (1.12)		.288^a^
Postpartum hemorrhage	3 (10.00)	458 (3.69)	1.809	.179
Anemia	9 (30.00)	2654 (21.37)	1.327	.249
Intrahepatic cholestasis of pregnancy	3 (10.00)	398 (3.20)		.071^a^
Gestational diabetes mellitus	3 (10.00)	2951 (23.76)	3.130	.077
Hypertensive disorders of pregnancy	3 (10.00)	924 (7.44)	0.034	.853
Cardiac insufficiency	0 (0.00)	20 (0.16)		1.000^a^
Uterine rupture	0 (0.00)	84 (0.68)		1.000^a^
D-dimer≥5.545 mg /L	21 (70.00)	3057 (24.61)	33.130	<.001
D-dimer≥6.475 mg /L	16 (53.33)	2480 (19.97)	20.789	<.001
D-dimer≥9.88 mg /L	11 (36.67)	1242 (10.00)	20.661	<.001

^a^ evaluated by Fisher’s exact test

A multivariate model using forward stepwise regression was constructed to identify the risk-factors associated with VTE in puerperium. D-dimer≥5.50 mg/L and emergency cesarean section were independently associated with VTE in puerperium (Table 3).

**Table 3:** Multivariate logistic regression of VTE risk factors during puerperium of women age ≥ 35

Risk factors	*P* value	OR	95% CI
Emergency cesarean section	.001	11.965	2.732–52.401
D-dimer≥5.545 mg /L	<.001	5.874	2.678–12.886

## Discussion

VTE was reported 1.0–1.8/1000 in women during pregnancy and puerperium [10]. Our study showed that the rate of VTE during puerperium of women age ≥ 35 (2.4‰) was higher than that of younger mothers, which supported age was a risk factor for VTE, which had been proved by many other studies [3, 11–13]. It was noteworthy that even among the women age ≥ 35, age was almost significantly different between the VTE and non-VTE groups (*p* = 0.058). Considering the limitation of sample size, age difference between groups was likely to be statistically significant if the sample size was enlarged. It was considered that more cases of DVT events occured in the left lower extremity, which was related to the more serious venous stasis of the left lower extremity caused by the compression of the pregnant uterus [14]. But in our study, the proportion of DVT in the left lower extremity was only a little higher than that in the right. This may due to the limitation of the small sample size.

The diagnostic value of D dimer for pregnant related VTE is not clear up to now. For pregnant women, D-dimer concentration increased progressively during the pregnancy and peaked at the first postpartum day [6]. Most healthy pregnant women have higher D-dimer values during pregnancy and puerperium than the normal reference range [15]. A prospective study showed that in the first trimester, 84% women had normal D-dimer, in the second 33%, and by the third trimester only 1%, which suggests that D-dimer has no practical diagnostic use of VTE if the threshold for abnormal is used [7]. Guidelines from Royal College of Obstetricians and Gynaecologists recommended that D-dimer testing should not be performed in the investigation of acute VTE in pregnancy [16]. Guidelines from American College of Obstetricians and Gynecologists also recommended that the rise of D-dimer cannot predict VTE reliably [7, 17]. However, there still some studies supporting to perform D-dimer test and provide a higher threshold to increase the specificity of D-dimer without reducing the sensitivity [7, 8, 18–20]. Actually, D-dimer test is still being used by obstetricians. If the D-dimer level was abnormally high, the need for prophylactic use of low molecular weight heparin (LMWH) was decided by the doctors according to their own experiences.

However, up to now, there are few studies on the correlation between D-dimer level and VTE in delivery women age ≥ 35. The prevention of VTE in delivery women age ≥ 35 is particularly important because the risk of VTE increases with age. A variety of acquired prothrombotic risk factors (e.g., autoimmune disorders, diabetes and infection) also gradually develop with aging. Concurrently, aging is associated with a variety of coagulation and hemostasis changes in general population [21]. In our study, a large sample of older mothers was observed and we initially found the predictive value of D-dimer test for VTE during puerperium in women age ≥ 35. Although the specificity and sensitivity are not particularly high, the ROC curve analysis offered an even higher threshold of D-dimer in delivery women age ≥ 35. Multivariate analysis also indicated that D-dimer≥5.50 mg/L was independently associated with VTE in puerperium of older mothers. Therefore, we think it is necessary to perform the D-dimer test within postpartum 24 h of women age ≥ 35.

Elevated D-dimer level was not the only criterion for high risk of VTE. Our study revealed emergency cesarean section was another important independent risk factors of VTE in puerperium of women age ≥ 35, which was consistent with previous studies. A meta-analysis found that the risk of VTE was four fold greater following cesarean section than following vaginal delivery, and was greater following emergency cesarean section than following elective cesarean section [4]. Other studies also revealed several independent risk factors of VTE in puerperium such as higher BMI, thrombophilia, multiple pregnancy, gestational diabetes, premature birth, anemia, chorioamnionitis, in vitro fertilization with ovarian hyperstimulation, cardiac diseases and postpartum hemorrhage [11]. But we didn’t find these risk factors because these factors may be age related and the objects of our study were older mothers while theirs were delivery women of all ages. Based on the results of this study, we recommend the use of LMWH to prevent VTE when the lever of D-dimer was higher than 5.50 mg/L of older mother, or the delivery mode of the older mother was emergency cesarean section.

The limitation of this study is that the sample size of VTE group is small, but this is consistent with the incidence of pregnancy-related VTE. Other limitations of this study include the effects of choice bias, for all the women in this study were at our hospital, and loss to follow-up bias, for we could hardly get the data of follow-up in communities and vascular department. Furthermore, this study did not discuss the predictive effect of D-dimer test for VTE during pregnancy and 24 h after postpartum, which need further study.

## Conclusion

In summary, this study calculated that D-dimer≥5.50 mg /L was an independent factor associated with VTE in puerperium of women age ≥ 35, which confirmed the predictive value of D-dimer test for older delivery women. Another independent risk factor of VTE in puerperium of women age ≥ 35 was emergency cesarean section. We believe our study provides a new reliable evidence for clinicians to focus on the emphasis risk factors for VTE of older delivery women, which was expected to reduce the incidence of VTE.

## Data Availability

Not applicable.

## Abbreviations

VTE: Venous thromboembolism
ROC: Receiver operating characteristic
BMI: Body mass index
CT: Computed tomographic
PE: Pulmonary embolism
DVT: Deep venous thrombosis
AUC: Area under the curve
LMWH: Low molecular weight heparin
